# Recent Advances in Astaxanthin Micro/Nanoencapsulation to Improve Its Stability and Functionality as a Food Ingredient

**DOI:** 10.3390/md18080406

**Published:** 2020-08-01

**Authors:** Óscar Martínez-Álvarez, Marta M. Calvo, Joaquín Gómez-Estaca

**Affiliations:** Institute of Food Science, Technology and Nutrition (CSIC), José Antonio Novais 10, 28040 Madrid, Spain; oscar.martinez@ictan.csic.es (Ó.M.-Á.); mmcalvo@ictan.csic.es (M.M.C.)

**Keywords:** liposomes, complex coacervation, spray-drying, solubility, antioxidant, anti-inflammatory, coloring

## Abstract

Astaxanthin is a carotenoid produced by different organisms and microorganisms such as microalgae, bacteria, yeasts, protists, and plants, and it is also accumulated in aquatic animals such as fish and crustaceans. Astaxanthin and astaxanthin-containing lipid extracts obtained from these sources present an intense red color and a remarkable antioxidant activity, providing great potential to be employed as food ingredients with both technological and bioactive functions. However, their use is hindered by: their instability in the presence of high temperatures, acidic pH, oxygen or light; their low water solubility, bioaccessibility and bioavailability; their intense odor/flavor. The present paper reviews recent advances in the micro/nanoencapsulation of astaxanthin and astaxanthin-containing lipid extracts, developed to improve their stability, bioactivity and technological functionality for use as food ingredients. The use of diverse micro/nanoencapsulation techniques using wall materials of a different nature to improve water solubility and dispersibility in foods, masking undesirable odor and flavor, is firstly discussed, followed by a discussion of the importance of the encapsulation to retard astaxanthin release, protecting it from degradation in the gastrointestinal tract. The nanoencapsulation of astaxanthin to improve its bioaccessibility, bioavailability and bioactivity is further reviewed. Finally, the main limitations and future trends on the topic are discussed.

## 1. Astaxanthin: A Valuable Marine Resource

### 1.1. Chemical Structure and Sources of Astaxanthin

**Figure 1:**
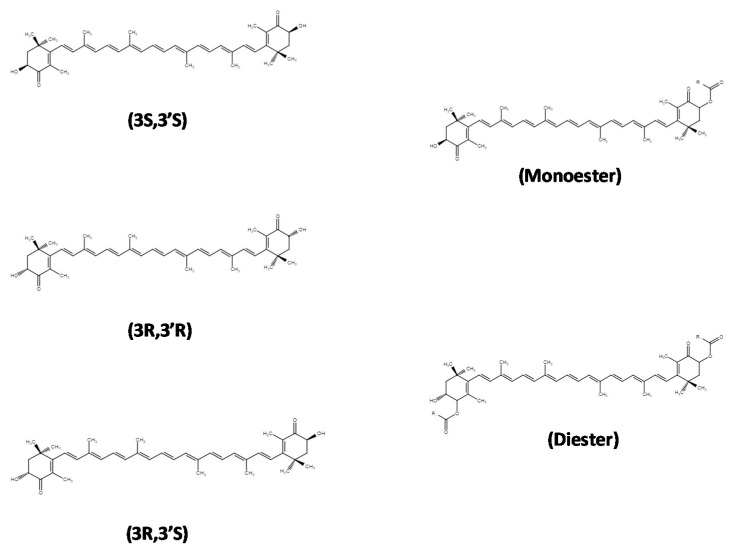
Configurational isomers of astaxanthin (enantiomers 3S, 3’S and 3R, 3’R and the mesoform 3R, 3’S) and monoester and diester forms (source ChemIDPlus)**.**

Astaxanthin (3,3′-dihydroxy-β,β′-carotene-4,4′-dione) is a carotenoid, which belongs to the group of xanthophylls responsible for the color of some plants, animals, and some types of microorganisms. Animals are unable to synthesize carotenoids, so they obtain astaxanthin through dietary intake. The astaxanthin molecule consists of two oxygenated ionone ring systems linked by a chain of conjugated double bonds (Figure 1). The β-ionone rings present two asymmetric carbons located at the 3, 3′ positions, and both a hydroxyl (–OH) and a carbonyl (C=O) group on either end of the molecule [1]. The double bonds exist in two geometric configurations: *cis* or *trans*—*trans* being the most predominant geometric form in natural astaxanthin [2]. Astaxanthin may also present three configurational isomers (Figure 1), varying from one organism to another [3]: two enantiomers (3S, 3′S), (3R, 3′R), and an optically inactive mesoform (3R, 3′S). The stereoisomers 3S, 3S’ and 3R, and 3R’ are the most abundant forms found in nature. Nonetheless, it is known that astaxanthin is produced by different organisms at different stereoisomeric ratios. In astaxanthin from crustaceans, three types of optical isomers can be found [1]. In contrast, synthetic astaxanthin consists of a racemic mixture of the isomers (3R, 3’R)/(3S, 3′S)/(3S, 3′S) at the stereoisomeric ratio of 1/2/1 [4]. The differences between isomers influence several properties related to the biological function, such as the antioxidant potential [5]. The different types of astaxanthin isomers in some fish and crustacean species and their distribution in the tissues have been reviewed by Yu and Liu [6].

Astaxanthin can be synthesized in nature or chemically. Natural astaxanthin is extracted from synthesizing organisms and microorganisms that are cultured or collected for this purpose or extracted from by-products of the fish industry (mainly from crustaceans). Some organisms that synthesize astaxanthin are the following: microalgae (*Haematococcus pluvialis*, *Chlamydomonas nivalis* [7], *Chlorella zofingiensis* [8], *Neochloris wimmeri* [9], *Scenedesmus acutus* [10]); bacteria (*Agrohacterium aurantiacum* [11], *Brevundimonas scallop* [12], *Paracoccus carotinifaciens* [13]); yeasts (*Xanthophyllomyces dendrorhous* [14]); Protista (*Aurantiochytrium* [15]; *Thraustochytrium* [16]) and plants (*Adonis annua* [17], *Adonis aestivalis* [18]). Since 1990, astaxanthin is synthetically produced and nowadays it is produced on a large scale, this being the most abundant method of production found in the world market (95% of the total) because of the lower cost of production (approximately 1000 dollars/kg) when compared with natural astaxanthin [5,19]. However, the use of petrochemical resources as raw materials in the production of synthetic astaxanthin, and the growing demand for natural products, have increased the search for natural sources of astaxanthin in recent years [19,20]. Thus, considerable efforts have been made to lower the production cost of natural astaxanthin, although the fact is that the production of natural astaxanthin from natural sources is nowadays limited [1]. The major producers of natural astaxanthin on an industrial scale are the algae *H. pluvialis* and the red yeast *X. dendrorhous* [21,22]. They are not very difficult to cultivate, and some conditions to increase the production of astaxanthin have been studied and applied in the industry. However, nowadays the interest in finding new methods to increase its production continues [23,24,25]. *H. pluvialis* can produce astaxanthin at >4% per dry weight (DW), which is favorable when compared with *P. carotinifaciens* (2.2% DW), *P. rhodozyma* (<0.5% DW) and shrimp/crab shells (<0.025% DW) [26]. Despite this, crustacean waste is a very relevant source of astaxanthin because the processing industry produces large amounts of cephalothorax and carapace residues (40–50% (*w*/*w*) in weight) containing astaxanthin [27]. The most common valorization option is probably obtaining chitin/chitosan, but other compounds of industrial interest, such as proteins, lipids, carotenoids, flavor compounds, or calcium carbonate, can be obtained as well [28,29].

The chemical composition, bioavailability, purity, and other characteristics of astaxanthin vary as a function of its origin [30]. In nature, astaxanthin is predominantly found esterified in the hydroxyl groups with one (monoesters) or two (diesters) units of fatty acids (Figure 1) or conjugated with proteins (carotenoproteins). These associations provide higher stability to astaxanthin, as compared with the non-esterified synthetic forms, which are very susceptible to oxidation [31,32]. The astaxanthin from *H. pluvialis* is constituted of 70% monoesters, 15–20% diesters, and 4–5% free forms [33], whereas *P. carotinifaciens* and *P. rhodozyma* produce astaxanthin in the free form [34]. Astaxanthin obtained from crustaceans is present in both esterified forms and as carotenoproteins. In this field, Gómez-Estaca et al. [35] reported 5 astaxanthin monoesters and 10 astaxanthin diesters in an astaxanthin extract obtained from Pacific white shrimp waste. The most frequent fatty acids in the esterified forms were docosahexaenoic acid (DHA, 22:6, ω-3) and eicosapentaenoic acid (EPA, 20:5, ω-3). Carotenoproteins from crustaceans have also been characterized [36].

The use of most of the methods proposed for astaxanthin extraction from natural sources allows for obtaining a lipid fraction containing astaxanthin, esterified or not, and other lipophilic compounds. This is due to the great number of compounds present in the natural source used that can be extracted concomitantly. Sometimes this is not a disadvantage because some of these compounds, such as other carotenoids, tocopherol, and polyunsaturated fatty acids, can have beneficial effects [35,37]. Astaxanthin can be extracted using different solvents such as methanol, petroleum ether, acetone, dichloromethane, and edible oils (e.g., sunflower oil, soybean oil, etc.) [38,39]. Supercritical carbon dioxide extraction has also been used to obtain astaxanthin [40,41]. The use of high pressure to improve the efficacy of the solvent during the extraction of astaxanthin has also been proposed [42], as well as ultrasound [43], microwave [44], magnetic-field-assisted extraction [45], pulsed electric field [46], microbial fermentation [14], and the use of enzymes [47].

### 1.2. Astaxanthin as a Food Ingredient

The use of astaxanthin and astaxanthin-containing lipid extracts as a food ingredient may have a double function. On the one hand, a technological functionality, as they present an intense red color that can provide foods with an attractive reddish color. Furthermore, their antioxidant activity would protect food during processing and storage, improving quality. On the other hand, astaxanthin and astaxanthin-containing lipid extracts may play a bioactive function when consumed, so they have great potential for the development of functional foods.

Considering the tendency towards the use of natural additives in detriment to synthetic ones and to the increasing consumer concern with the diet-health relationship, astaxanthin extracts present great potential as food ingredients. However, some intrinsic properties constrain the use of astaxanthin and astaxanthin-containing lipid extracts as food ingredients, such as instability, low solubility (limiting dispersion in food matrices, bioaccessibility and/or bioavailability), difficult dosage and manipulation, and, in the case of crustaceans or algae extracts, intense odor and flavor [48,49,50,51,52,53,54,55,56,57,58]. Encapsulation involves the coating or entrapment of a pure material or a mixture (known as a core material or active compound) into another material (called the capsule, wall, or shell). It provides a physical barrier between the core compound and the surrounding media, giving rise to a wide range of potential applications in the food, agrochemical, and pharmaceutical sector. Encapsulation may help to overcome most of the problems that astaxanthin and astaxanthin-containing lipid extracts may present to use them as food ingredients, namely:

- Enhancing the organoleptic characteristics of the food product by masking undesirable flavor and odor [59].
- Facilitating the handling and dosage by converting a lipid extract or oleoresin into a powder, and also by a dilution effect in the wall material [58,60].
- Increasing solubility and dispersibility in food matrices, thus improving coloring capacity [57,61], bioaccessibility, and bioavailability [53,58,62].
- Improving cell membrane transport, which also improves bioavailability [63].
- Improving bioactivity, derived from increased solubility, bioavailability, and/or cell membrane transport [64,65].
- Protecting from degradation or loss of functionality due to the effects of thermal treatment, light, oxygen, pH, moisture, or interaction with food matrix components [66,67].
- Favoring the release at controlled rates or under specific conditions [61,68,69,70].

This review provides an overview of the use of different micro/nanoencapsulation techniques for improving the stability, functionality, and bioactivity of astaxanthin. This factor is crucial to use astaxanthin as a functional ingredient in food.

## 2. Insights into the Selection of Encapsulation Method and Wall Material for Astaxanthin and Astaxanthin-Containing Lipid Extracts

Encapsulation methodologies are diverse, originating in particles of different sizes (microparticles, nanoparticles) and structures (simple, multiwall, irregular, multi-core, matrix systems) [71]. Encapsulation techniques can be classified as chemical/physico-chemical or physico-mechanical methods. There is a wide variety of encapsulation methods, such as spray drying, spray chilling, extrusion coating, fluidized bed coating, liposome entrapment, coacervation, inclusion complexation, interfacial polymerization, solvent evaporation, solvent diffusion, nano-precipitation, salting-out, ionic gelation, or electrohydrodynamic atomization [71,72,73]. Most of them have been either applied to the encapsulation of astaxanthin or their use is feasible. The size of the capsules is an important factor to be taken into account, as structures on the nanometric scale (usually below 100 nm) present a vast range of novel physico-chemical characteristics different from those of bulk or micro-scaled substances. The large surface area per unit mass of nano-sized biomaterials may increase their functionality, biological activity, or allow the release of compounds at controlled rates [74]. The structure also has a relevant influence, as structures composed of various layers generally present higher stability and a more sustained release pattern [51,75].

Regarding the type of wall materials, food-grade biopolymers must be used for food applications; these include carbohydrates, gums, proteins, and lipids [71,76]. Factors to be taken into account, regarding their use, are their technological properties (solubility, film-forming ability, emulsifying capacity, hygroscopicity, viscosity, gelling ability, melting point), physical state (amorphous, glassy), organoleptic properties (color, odor, taste), and physico-chemical compatibility with the core material [52,75]. In this connection, it is common to use surface-active compounds as emulsifiers to improve compatibility [58]. The use of complexes from various polymers is a promising strategy, as they may show different functionalities from the plain components [77]. These factors, as well as the type and size of the structure, will determine the functionality, release kinetics, and stability of astaxanthin, and, in consequence, the potential applications of the ingredients developed.

The selection of the encapsulation method and wall material is not an easy task and will depend on many factors, for example:

- The time of storage of the ingredients developed until use—that is, if a special need for stability is required.
- The type of food product to be applied: the necessity of masking undesirable odor and taste; the necessity of protection from aggressive conditions (low pH, light or oxygen exposure, thermal treatments); food products with long shelf life (stability needs).
- The specific functionality to be improved: solubility, coloring capacity, bioavailability, antioxidant activity, etc.
- Aspects related to food formulation: water content, inclusion level, loading capacity, compatibility of raw materials, etc.
- Availability and costs of raw materials and equipment, processes, logistics, etc.

In the following section there is a brief discussion on relevant aspects regarding the selection of the encapsulation method and wall material is presented.

### 2.1. Encapsulation Methods to Improve Stability of Astaxanthin

Generally speaking, as astaxanthin is a thermolabile compound, encapsulation methods that avoid heating would be desirable. For instance, spray-drying has been found to induce astaxanthin losses, which ranged from 30 to 40% [58,78,79]. Despite this, spray-drying is a rapid and cost-effective encapsulating method widely employed in the industry [80]. Although high stability would be desirable in any case, it is especially relevant in the case of ingredients that need to be stored for long periods before use or if they are to be applied to foods in which degradation boosting (low pH, thermal treatments, presence of oxygen) or strong interactions with matrix components are expected. Multilayer structures—for example, those based on liposomes or oil-in-water emulsions—have shown greater physical stability and astaxanthin protection, as compared with uncoated or monolayer structures [51,81,82].

Encapsulation efficiency is a relevant factor regarding stability, as surface astaxanthin is readily degradable. Surface-active biopolymers, such as gum arabic or proteins, generally improve encapsulation efficiency, as compared with polysaccharides such as inulin or maltodextrin [52,60]. Regarding lipid-based systems, the use of mixtures of lipids with different melting points (liquid and solid) diminishes recrystallization phenomena and astaxanthin migration to particle surfaces [52,83]. The systems developed under this concept are referred to as nanostructured lipid carriers. Due to the excellent barrier to the moisture of lipid wall materials [83], these developments would be desirable for applications to moist foods with long shelf life periods to protect against chemical/light degradation or oxidation. Nevertheless, the possible oxidation of the wall material should also be considered, as it may induce quality loss of the food product [84].

A comprehensive evaluation of processing costs (equipment acquisition, energy input, raw materials), loses during storage as a function of encapsulation method and storage conditions, functionality of encapsulated ingredient, and desired food application should be taken into account to select the most appropriate encapsulation method [59,78].

### 2.2. Importance of the Water Content in the Incorporation of the Capsules in Foods

The encapsulation method to be used will depend on the water content of the food to be further incorporated. Thus, the use of micro- or nanoemulsified systems (including micro/nanoemulsions, fresh liposomes/nanoliposomes, solid lipid nanoparticles, nanoparticulate lipid systems, etc.) is much more restricted in foods with low or intermediate moisture levels, as well as in reformulated humid foods such as pâtés, sausages or surimi-based products in which moisture adjustment is crucial to modulate their textural properties [85]. Encapsulation methods, such as spray-drying or electrohydrodynamic atomization, among others, which produce dry micro- or nanoparticles, would be preferable in those cases. For some encapsulating methods that yield micro- or nanoparticles dispersed in water or other solvents (e.g., coacervation, solid lipid nanoparticles, or liposomes), the application of subsequent separation and drying steps is possible, but the additional cost ought to be assumed. Furthermore, the functionality of the reconstituted powders must be evaluated, due to possible modifications after drying [56,86,87]. The presence of high water content in such systems also has implications at the logistic level in case the ingredients need to be stored or transported.

### 2.3. Importance of the Encapsulation to Improve the Functionality and the Bioavailability of Astaxanthin

For applications, such as coloring, a homogeneous dispersion in the food matrix is mandatory, for which a good solubility/dispersibility and compatibility between the wall material and the food components are needed. Biopolymers processed by various methods—e.g., spray-drying or complex coacervation—have shown good compatibility with moist food matrices, such as gelatin, gelled fish products, or yogurt, providing homogeneous color and/or improving antioxidant activity or bioavailability [57,59]. Lipid-based carriers would be initially more indicated for applications in fatty foods due to the chemical compatibility; however, nanostructured lipid-based carriers containing astaxanthin have also shown good dispersibility in water-based products, such as surimi-based foods or beverages [56,84,88], pointing to the great potential of nanotechnology in the encapsulation field, as it improves dispersibility and stability due to size reduction. For applications in which the odor or flavor of the core material is not desirable—e.g., in fruit juices, milk products, etc.—insoluble micro- or nanoparticles with high encapsulation efficiency are required—for example, with those formed by complex coacervation [59].

Focusing on the use of astaxanthin as an ingredient for the design of functional foods, the increase in its intrinsically low bioavailability is crucial. This effect may be associated with: increased bioaccessibility (higher astaxanthin content in the gut lumen available to be absorbed), which is generally related to astaxanthin solubility [58]; increased absorption, mainly related to astaxanthin intake by gut epithelial cells [62]; a combination of both. Furthermore, the release kinetics of astaxanthin from the carrier system should be taken into account, considering the wall solubility and susceptibility to digestive enzymes [52]. Proteins release astaxanthin relatively quickly due to their water sensitivity and susceptibility to digestive proteinases, whereas lipid carriers allow a prolonged release by their slow disintegration and solid structure [52,83]. Acid-stable biopolymers, such as alginate, protect astaxanthin during gastric digestion [89]. However, the physical stability of solid lipid nanocarriers to gastrointestinal digestion depends on the lipid and surfactant composition [90]. The combination of lipid nanocarriers coated with gastrointestinal-resistant biopolymers, such as the combination of oxidized dextran and bovine seroalbumin, has also been proposed as a means to take advantage of both wall material families [68]. Other factors to be considered are particle size (smaller ones generally present higher bioaccessibility) and charge (positively charged ones can be adhered to enterocytes, promoting cell transport). The ability to form micelles also determines bioavailability; in this sense, emulsified lipid systems (emulsions, solid lipid nanoparticles, nanostructured lipid carriers, or liposomes) are generally considered good vehicles to promote bioavailability [83].

### 2.4. Use of the Encapsulation to Modulate the Release Rate of Astaxanthin

The need for specific release conditions or specific release rates is another factor affecting the selection of the encapsulation method and the wall material. Drug release from a polymer matrix can be categorized in three main processes or systems [91,92,93,94,95,96,97,98,99,100,101,102]: (1) diffusion from the non-degraded polymer (diffusion-controlled system); (2) enhanced drug diffusion due to polymer swelling (swelling-controlled system); (3) release by polymer degradation and erosion (erosion-controlled system). Generally speaking, the release velocity will follow the order 3 > 2 > 1. The release kinetics of astaxanthin from a polymeric matrix by diffusion depends, as for any other molecule goes, on its solubility and diffusivity through the different compartments involved—core material, microcapsule layer(s) and/or surrounding media (food). For example, astaxanthin encapsulated in a lipid-based system will generally be released by simple diffusion, the velocity depending on the chemical compatibility with the surrounding media (more quickly in oily than in aqueous foods). Strategies to slow down diffusion may include designing encapsulating systems composed of multiple shells or increasing the degree of reticulation of the matrix, as reported by Lee et al. [70]. An example of enhanced drug diffusion due to polymer swelling would be encapsulation in a moisture-sensitive biopolymer that swells upon hydration (e.g., protein, carbohydrates, or gums), thus boosting release due to an increase in free volume, facilitating astaxanthin diffusion. Furthermore, this represents a strategy to trigger the release, as astaxanthin would be relatively stable inside the dry capsule until hydration. A potential application is, for example, to powder mixtures rendering soups or beverages after reconstitution in water [56,57]. In an erosion-mediated system, the wall needs to be totally or partially solubilized or degraded—for example, in a water-soluble biopolymer, such as gelatin or maltodextrin applied to moist food, or a fatty wall applied to a food product subjected to a temperature over its melting point. This is also the main release mechanism in the gastrointestinal tract due to the action of proteolytic and lipolytic enzymes, prompting release.

Keeping all this in mind, factors affecting the release rate to which attention must be paid are the type of polymer employed (considering its modification—e.g., by chemical treatments or by using hybrid materials), the type of encapsulation method, with special emphasis on the use of multiple walls (they add additional barriers to diffusion), or the size of the micro/nanocapsules (initially the lower the size, the faster the release), and finally the type of food to be applied to or the processing to which the food is being subjected (e.g., thermal treatment, etc.).

## 3. Encapsulation Technologies to Improve the Technological Functionality of Astaxanthin and Astaxanthin-Containing Lipid Extracts: Main Achievements

### Effect of the Encapsulation on the Technological Functionality and Stability of Astaxanthin

The main potential technological applications of astaxanthin and astaxanthin-containing lipid extracts as food ingredients are as red-coloring and antioxidant agents [57]. Applications of encapsulation technologies to improve these functionalities are generally related to the increase in astaxanthin solubility, improving its dispersion and accessibility to the water fraction of the food. Furthermore, the improvement of astaxanthin stability both during storage and once incorporated into a food product is crucial to use it as a food ingredient. Applications of encapsulation technologies to reduce the sensory impact of astaxanthin-containing lipid extracts have also been reported. These are the aspects that will be discussed in this section, with special emphasis on practical applications of encapsulated astaxanthin and astaxanthin-containing extracts in foods. Two summary tables have been constructed to illustrate the extension of the work published on this topic. Table 1 summarizes some articles related to the encapsulation of astaxanthin and astaxanthin-containing lipid extracts, giving information on the encapsulation methods, wall materials used, and main achievements concerning the improvement in its technological properties or stability. In Table 2, the main applications to food models or food products, along with the main achievements, are summarized.

Kim et al. [66] developed inclusion complexes of astaxanthin and β-cyclodextrins, achieving a solubility increase in astaxanthin by 110-fold, as well as an improvement in stability against heat, pH, UV light and oxidation by 7–9-fold, as compared with free astaxanthin. The main drawbacks of this ingredient are that pure astaxanthin needs to be used for proper inclusion complex formation and that the astaxanthin:β-cyclodextrin optimum ratio to form the complexes is very high (1:200), thus hindering its applications in the food sector. On the other side, it is spray-drying, which is a widespread cost-effective drying method in food industries that have been assayed for the encapsulation of astaxanthin extracts from both microalgae or shrimp by-products with good results at improving functionality [57,58,60,78,79,92,93,94,95]. This process also has the advantage, as compared with cyclodextrin inclusion complexation, of upgrading the whole ingredient, avoiding any additional separation steps. Astaxanthin extracts from microalgae and shrimps also contain polyunsaturated fatty acids and/or tocopherols with beneficial properties [35,96].

Some factors affect the properties of spray-dried microcapsules and their functionality, including processing parameters (temperature, flow rate, airflow, etc.), wall materials, core material composition, wall-core ratio, etc. [80]. The scope of the review is not to discuss the effect of these parameters but to focus on the improvement of the technological properties of the extracts to be used as food ingredients. Montero et al. [58] encapsulated a lipid extract from shrimp waste by spray-drying and employing different wall materials rendering powders of high water solubility (>92%). Although thermal treatment caused lipid oxidation and astaxanthin loss to a certain extent, further astaxanthin loss was not observed during chilled storage (110 days) and lipid oxidation was low, pointing at the stability of the ingredients developed. Ahmed et al. [78] also observed the loss of astaxanthin when spray-drying an extract from *H. pluvialis*, whereas freeze-drying rendered a higher yield of astaxanthin due to the absence of thermal treatment. Zho et al. [53] found a reduction in astaxanthin loss during storage thanks to encapsulation by complex coacervation, as compared with free oleoresin, finding an inverse relationship between storage temperature and stability. This explains the high stability of spray-dried encapsulated astaxanthin found by Montero et al. [58] during chilled storage. A possible strategy to reduce the lipid oxidation derived from spray-drying or storage is the appropriate selection of wall materials, as well as the addition of antioxidants [94].

Improvements in astaxanthin stability against detrimental factors, such as light, oxygen, oxidants such as Fe^3+^, pH, or thermal treatments were also found by using other encapsulation methods during in vitro assays (Table 1) [48,49,50,51,52,53,54,55,56,57,59,61]. Comparing works is quite difficult because of the variety of encapsulation methods, wall materials, astaxanthin extract origin, and assay conditions and methodologies; however, the amount of work published demonstrates the efficacy of encapsulation technologies to partially protect astaxanthin from external detrimental factors. In this sense, the application of multilayer structures appears as a promising strategy to improve astaxanthin stability. Qiang et al. [51] and Pan et al. [81] developed chitosan, lactoferrin or whey protein isolate-coated liposomes which showed an improvement of physical stability during storage or thermal treatment, reducing leakage and/or the thermal or light-induced degradation of astaxanthin, as compared with uncoated liposomes. Similar results were obtained by Liu et al. [82], who found that multilayer-coated emulsions improved the chemical stability of encapsulated astaxanthin, as well as the aggregation stability of the lipid droplets at elevated ionic strengths and temperatures. Regarding applications in food products, encapsulated astaxanthin extract from *P. rhodozyma* by a modified emulsification-evaporation process has shown astaxanthin retention after 4 weeks of storage at 25 °C when added to liquor, rice or apple vinegar [48]. In another study, spray-dried encapsulated shrimp extract was incorporated into the formulation of cookies, and no marked changes in EPA, DHA, and astaxanthin contents were noticeable after 12 days of storage even in the presence of light [97]. Gómez-Guillén et al. [57] evaluated the suitability of spray-dried lipid extract from shrimp as a technological ingredient for aqueous food applications and found a high solubility of the powder in water. Furthermore, irrespective of dissolution temperature (20 °C/100 °C) or refrigerated storage time of reconstituted powders (up to 6 days at 5 ± 1 °C), the color, astaxanthin content, and fatty acid composition of the encapsulated lipid extract were very stable.

Astaxanthin water solubility is a key factor that influences not only the coloring capacity and antioxidant activity of food, but also the bioavailability when consumed. Many encapsulation methods have rendered very good astaxanthin solubility/dispersibility improvements, such as β-cyclodextrins complexation, spray-drying, ionotropic gelation, nano-precipitation protein complexation, liposome entrapment, or emulsification-evaporation [48,57,61,66,77,98,99]. They mostly employ soluble biopolymers, such as proteins, gums, or modified polysaccharides, that specifically interact with astaxanthin, as in the case of some proteins [77] or form stable emulsions in water [79]. Lipid-based nanostructured systems, such as nanoliposomes, solid lipid nanoparticles, or nanostructured lipid carriers also improve dispersibility, owing to their small particle size [61,83,100]. These systems scatter light weakly and, therefore, can be incorporated into optically transparent beverages; furthermore, they are generally very stable to particle aggregation and gravitational separation [100]. Gómez-Guillén et al. [57] found a good dispersion of spray-dried encapsulated shrimp lipid extract in an aqueous food matrix, such as a gelatin gel, providing a uniform and attractive red color. Similar results were found by Zhu et al. [56] in a beverage food model, Jiang and Zhu [48] in vinegar and liquor, Gulzar et al. [88] in milk, and Marín et al. [87] in a surimi-based product. However, the increase in solubility is not always a requirement to obtain a good dispersibility and coloring capacity, as other methods rendering insoluble microcapsules or beads, such as complex coacervation or ionotropic gelation, have also shown good results, as compared to free astaxanthin—e.g., in yogurt and gelled fish product [59,101].

Antioxidant activity is concomitantly improved with water solubility as a result of encapsulation by different technologies (Table 1) [48,50,57,64,68,70,87,98,99,102]. Furthermore, encapsulation prevents the loss of antioxidant activity during the storage of the ingredients [70]. Despite the high antioxidant potential of encapsulated astaxanthin, the applications of these ingredients in the prevention of lipid oxidation in food products are scarce. On the contrary, the in vivo effects are well studied and discussed in the following section. Marín et al. [84] entrapped a shrimp lipid extract containing astaxanthin in nanoliposomes and evaluated the effect of their incorporation into a surimi-based product after freeze-drying. Although they found an improvement in antioxidant activity due to encapsulation, the incorporation of the ingredient harmed the oxidative stability of the products developed during frozen storage. The authors attributed it to the unsaturated nature of fatty acids in phosphatidylcholine, which may have been prompted during liposome formation; however, the contribution of the lipid oxidation of the shrimp extract cannot be discarded. In this connection, Takeungwongtrakul and Benjakul [97] found increased lipid oxidation in biscuits that incorporated spray-dried encapsulated shrimp lipid extract, owing to the oxidation of its unsaturated fatty acids. Feng et al. [50] evidenced the increase in antioxidant activity of yogurt with incorporated encapsulated astaxanthin extract from *P. rhodozyma*, but they did not perform a study on its oxidative stability. In another work, shrimp lipid extract encapsulated in nanoliposomes was added to skim milk and no significant lipid oxidation was observed after 15 days of chilled storage [88]. These works, although scarce in number, point at the complexity and the number of factors to be considered with a view to using astaxanthin extracts as food antioxidants; more work should be performed for a proper evaluation of its performance.

Masking the undesirable sensory properties of astaxanthin-rich lipid extracts is very interesting in the development of functional foods so that therapeutic doses could be incorporated in the food without sensory impairments. Gulzar and Benjakul [103] showed the suitability of nanoliposome encapsulation to reduce the fishy odor of the shrimp extract during storage through a trained sensory panel. Indeed, the ability of nanoliposomes to retard the release of astaxanthin in aqueous media has been previously reported [5,15]. In other works, complex coacervation has also shown its suitability for masking fishy odor and/or the odor of shrimp extracts, depending on the wall materials employed and the foods to be applied [59,86]. The complex formed by gelatin and gum arabic is more appropriate than the gelatin-cashew gum to mask the sensory characteristics or shrimp extract when added to yogurt, probably due to the higher encapsulation efficiency. When applied to a gelled fish product, the gelatin-gum arabic complex failed at masking the fishy flavor and odor, probably due to capsule disintegration during thermal treatment for gel formation [104]. In another work, in which spray-dried microcapsules containing a shrimp extract were applied to the formulation of biscuits, the optical properties (measured by the objective measure of color and sensory panel) were affected to a lower extent than when comparable amounts of free astaxanthin were added. However, as an untrained sensory panel was used, the ability of encapsulation to mask shrimp-odor could not be evaluated. Despite this, a good sensory rating was achieved at the intermediate inclusion level assayed.

## 4. Effect of the Encapsulation on the Bioactive Properties of Astaxanthin

Several health-related benefits have been described for astaxanthin, including anticancer activity, cardiovascular disease prevention, anti-diabetic activity, anti-ischemic effect, neuroprotective properties, anti-allergic activity, and the enhancement of the immune response, among others [105,106,107,108]. Although the mechanism by which astaxanthin seems to exert functional effects is still unknown, it is thought that some are related to its intense antioxidant capacity, greater than that of vitamin E, and other carotenoids, such as zeaxanthin, lutein or β-carotene [109,110,111,112]. These potential health-promoting properties, together with the ability to cross intestinal mucosa and reach plasma and target tissues [105], make astaxanthin a promising novel functional ingredient to be incorporated in foods. Although astaxanthin is widely commercialized as a nutritional supplement, the reality is that shreds of evidence of its health effects have been observed mainly in animal models and not in humans [113,114,115,116,117,118,119], this being a crucial point in the development of functional foods.

It is known that poor bioavailability limits the biological functionality of astaxanthin. Thus, when astaxanthin is administered orally, the bioavailability ranges from 10–50% of the given dose [120]. The poor bioavailability of astaxanthin is mainly attributed to its low water solubility which will hinder its absorption in the intestine. Interestingly, the esterified state of natural astaxanthin [35] enhances the bioavailability by easing the incorporation into mixed-micelles in the lumen, where the unsaturated lipids are released by the action of biliary salts and pancreatic lipases before the cellular uptake of free astaxanthin by intestinal mucosal cells [105,121].

The low bioavailability and stability of astaxanthin leads to scarce accumulation in target organs and might suggest that it should be incorporated in excess in functional foods to induce a biologically relevant effect. However, high concentrations of astaxanthin could compromise the physico-chemical and sensory characteristics of food. High doses of astaxanthin orally administered could also have certain adverse effects on human health, as it shows high reactivity and easily switches from antioxidant to pro-oxidant. Factors such as excessive concentration, oxygen pressure, and interaction with other redox agents may trigger this change, as observed by different authors [110,122]. The oxidation products formed may produce oxidative damage in DNA, lipids, and proteins in cells, and modulate gene expression, leading to negative consequences in human beings [121]. Using natural extracts instead of synthetic astaxanthin is recommended due to the presence of natural antioxidants, such as α-tocopherol, that would protect it from autooxidation [35,123]. Nonetheless, it is important to note that the pro-oxidant effect of astaxanthin could be of interest to induce tumor cell damage by enhancing ROS generation [122].

### 4.1. Encapsulation to Improve the Oral Bioavailability of Astaxanthin

To incorporate astaxanthin as a functional ingredient in foods, it is important to consider that its poor bioavailability is also attributed to its low physico-chemical stability when exposed to external agents as light, extreme pHs, oxygen, and heat. Hence, industrial processing will presumably negatively affect the potential health effect of food-containing astaxanthin [107]. Other factors, such as the interaction with other compounds in the food matrix, will limit the bioavailability of astaxanthin [110]. The stability and bioavailability of astaxanthin could then be improved by loading it in micro/nanoparticles made with edible matrices before being incorporated in functional foods. In this research field, different lipid-based, organic, or inorganic-based and hybrid micro and nanoparticles, more or less complex, have been designed for improving the stability and bioavailability of astaxanthin [52]. Among them, the coacervates and the lipid-based micro/nanoparticles are the systems that have attracted the most attention in food industries [83,124], mainly due to the possibility to be scaled up.

Despite the great potential of astaxanthin carriers in the sector of functional foods, there is scarce information on delivery systems with the ability, in vivo, to protect astaxanthin against adverse environments and target at the intended biological site where it can be released in a controlled manner and at the desired time. To get to this point, much attention must be paid to the stability and release from the food matrix of the astaxanthin-loaded particles within the gastrointestinal tract. In this regard, the encapsulation of astaxanthin in lipid-based micro/nanoparticles improves the bioaccessibility and bioavailability of astaxanthin [125]. These particles are composed of one or more lipid bilayers and can protect astaxanthin against gastrointestinal digestion, stimulate the secretion of bile acids and pancreatic lipases that will facilitate lipid digestion and astaxanthin release. The free astaxanthin could then be solubilized into mixed micelles and be absorbed by passive diffusion or by specific epithelial transporters in the intestinal epithelium [83]. The composition and the physico-chemical and colloidal properties of the lipid-based particles will influence the bioaccessibility and bioavailability of astaxanthin, as well as the presence of specific components in foods, such as soluble dietary fiber, which compromise astaxanthin absorption [52,105,124]. Liposomes seem to be less resistant to degradation than nanostructured lipid carriers in the presence of bile acids and pancreatic lipases. The stability and, consequently, its half-life could be enhanced by various mechanisms, such as the use of saturated lipids, or the simultaneous use of phytosterols and phospholipids [83]. Surfactant-based delivery systems (niosomes) have also been proposed for their resistivity in acid media and against enzymatic hydrolysis [83]. However, it is important to note that both liposomes and niosomes show low stability when dispersed in a liquid media, thus their incorporation in liquid foods would not be recommended.

Other materials have also been used to protect astaxanthin against gastrointestinal digestion. Among them, alginate stands up, as it is insoluble in gastric acidic pH, which prevents degradation, but allows dissolution at intestinal pH where the encapsulated compounds are released [89,102]. Li et al. [67] encapsulated astaxanthin oleoresin in alginate/gelatin microcapsules by coacervation and evaluated the stability of the microcapsules and the astaxanthin release under in vitro digestion. They observed resistance of the microcapsules under gastric digestion, and a burst release of astaxanthin within the first 4 h of intestinal digestion (more than 60%) because of the rapid degradation of the microcapsules under mild alkaline conditions. The authors reported an increment of bioavailability in mice, showing the highest plasma concentration of free astaxanthin after 2.5 h of gastric administration. Zhou et al. [53] compared the potential bioavailability of astaxanthin oleoresin with that of esterified astaxanthin encapsulated in whey protein/gum Arabic coacervates. Both were subjected to simulated gastrointestinal digestion, and the amount of free astaxanthin at the end was significantly higher when astaxanthin was protected, this being ascribed to the presence of whey protein and gum Arabic, which facilitated the activity of pancreatic lipases by the generation of lipid-water interfaces. A further in vivo experiment with mice gavaged with the microcapsules confirmed an increment of concentration of free astaxanthin in plasma. Unlike the results observed by Li et al. [67], the maximum plasma astaxanthin concentration was reached 9 h after the administration of the microcapsules. The differences between both studies are ascribed to differences in the controlled release rate of astaxanthin in the digestive tract, suggesting that the microcapsules made with whey protein/gum Arabic show more mechanical strength under intestinal conditions than those prepared with alginate/gelatin, which were more susceptible to degradation or erosion. Zhou et al. [53] suggested that the microcapsules were degraded by mechanical forces, resulting from gastrointestinal peristalsis. Thus, these works lead us to think that the selection of the blends used to prepare the coacervates is important to modulate the intestinal release of astaxanthin, being essential not only to resistance to the environment in the intestine, but also the mucoadhesive properties that will delay gastrointestinal transit, and hence permit the retention of the microcapsules until degradation. Additionally, the particle size is important in the transit rate, since it is slower for the smallest microcapsules.

The role of some delivery systems is not only limited to the protection of astaxanthin against gastrointestinal digestion and later release in the intestine. Some astaxanthin loaded-carriers, lower than 500 nm, could maintain their integrity and be absorbed through Peyer’s patches or by endocytosis, and thus enhance the bioavailability of astaxanthin [126,127]. In this regard, the composition, size, charge, and surface of the nanoparticles used to load astaxanthin play an important role. Recently, Hu et al. [98] have developed complex chitosan-casein-oxidized dextran nanoparticles to enhance the oral availability of astaxanthin. These particles (120 nm and yield of 70%) improved the dispersibility of astaxanthin and were stable in simulated gastrointestinal fluids. The use of chitosan-based nanoparticles to load astaxanthin presents the advantage that chitosan is generally recognized as safe (GRAS), biodegradable, exhibits low toxicity, and can improve the transport of bioactive molecules through the epithelial tight junctions because of its affinity to the cell membrane [128,129]. However, nanoparticles only made with chitosan will not protect astaxanthin during gastric digestion, as it is degraded under low pH values [129], hence, to use chitosan, it is necessary to blend it with nonionic polymers to improve the physico-chemical stability of the particles. Nanoliposomes below 200 nm size have been also suggested as good astaxanthin carriers across the intestinal barrier because of their lipophilic nature and their ability to adhere to membranes and penetrate cells [124]. The amount of phosphatidylcholine greatly affects the cell uptake efficiency and high amounts in the liposome (70%) were recommended, instead of lower amounts (23%), which did not show a significant uptake in Caco-2 monolayers.

### 4.2. Evidence of Biological Effects of Encapsulated Astaxanthin in Cell Culture and In Vivo

Some of the works revealing the bioactive functionality of encapsulated astaxanthin in vitro or in vivo are summarized in Table 3. The free astaxanthin released by the carrier in the gut is absorbed by the intestinal epithelium and associates with lipoprotein particles, such as chylomicrons, LDL, VLDL, and IDL, along with other lipids. Then, it will be stored and eventually metabolized in the liver [110]. Free astaxanthin could reach target tissues and incorporate in biological membranes and scavenge reactive species, this being crucial in the antioxidant effect, as observed in cultured cells [130]. Unlike other carotenoids, which orient into membranes parallel to the membrane surface, the presence of polar and nonpolar regions in the structure allows astaxanthin to insert into the lipid bilayer, with the polar end groups oriented toward the polar hydrophilic headgroup region of the lipid membrane [131]. This different orientation strengthens the membrane and allows astaxanthin to scavenge free radicals generated at the surface of membranes or even inside, protecting lipids in the bilayer against peroxidation. Peng et al. [63] postulated that astaxanthin could even pass through the cell membrane by passive transport and reach the cytoplasm in a process that may depend on the membrane characteristics (cholesterol/phospholipid ratio), affinity, and/or dissolution rate.

The nanoparticles with the ability to pass through Peyer’s patches protect astaxanthin against hepatic metabolism in the liver [127] and reach target organs by the lymphatic system. If nanoliposomes are used, their lipophilic nature also facilitates the adherence to the cell membrane and hence the cellular uptake, as observed by Sangsuriyawong et al. [124] in Caco-2 cells. Similar results were observed by Peng et al. [63] in the liver cancer cell lines, HepG2 and Hep3B, in which the nanoliposomes (251 nm size) were able to slowly deliver astaxanthin into the cytoplasm. Astaxanthin then induced apoptosis by the induction of subG1 detention and increased the activity of three antioxidant enzymes: superoxide dismutase (SOD), catalase, and glutathione S-transferase. These effects would be ascribed to the ability of astaxanthin to activate signaling pathways able to modulate gene expression. In this sense, the antiproliferative effect of astaxanthin has been suggested to be produced by the suppression of inflammatory cytokine expression and triggering of immune-system specific gene expressions [122].

Other promising nanoparticles have proven to be suitable for the intracellular delivery of astaxanthin, some of these being made with other edible matrices (Table 3). Montanari et al. [132] tested the antioxidant effect of natural astaxanthin loaded-hyaluronan-based nanohydrogels in oxidatively stressed human vein endothelial cells (HUVEC cells). They observed that the astaxanthin loaded-nanohydrogels (287 nm average size, Z potential around 40 mv) showed a similar ability to scavenge free radicals in vitro as that of free astaxanthin. The nano-formulations were taken up by the cells and accumulated in the perinuclear area and were able to neutralize Reactive Oxygen Species (ROS) without showing any cytotoxicity. Zuluaga et al. [133] performed a similar experiment using a hydroxypropyl-beta-cyclodextrin-astaxanthin complex, observing an increment in the solubility of astaxanthin and an important antioxidant effect mediated by the Nrf2/HO-1/NQO1 pathway. These authors noticed that the complex was able to upregulate the Nrf2 gene expression.

Wang et al. [128] used DNA and chitosan to encapsulate astaxanthin by macromolecular co-assembly. The nanoparticles (average size of 211 nm and polydispersity index of 0.29) showed stability under light red color, were well dispersed in water, and exerted an important cytoprotective effect on Caco-2 cells after the induction of oxidative stress with hydrogen peroxide when compared with astaxanthin. This effect is ascribed to the ability of the nanoparticles to be quickly absorbed through endocytosis by the cells, this being facilitated by their positive surface charge, as it enhances the permeability of cell membranes and hence, the cellular uptake efficiency of the particles. These nanoparticles did not show any cytotoxicity and were able to scavenge, with high efficiency, reactive oxygen species (ROS) in these H2O2-treated Caco-2 cells. Cellular uptake of these nanoparticles was also demonstrated in L929 fibroblasts. However, it is important to note that the cellular uptake efficiency was affected by the interactions between the nanoparticles and the extracellular matrix of the cell lines. Hu et al. [98] observed, in hepatic stellate cells LX-2, that chitosan-casein-oxidized dextran nanoparticles loaded with astaxanthin decreased the expression of two fibrogenic genes induced by the cytokine TGFβ1: the ACTA2 gene, which regulates the production of smooth muscle α-actin, and the COL1A1 gene, which regulates that of type 1 procollagen, alpha 1 [137]. The levels of both proteins, likewise, decreased when compared with the effect of unencapsulated astaxanthin. The results obtained in this study suggest that the nanoparticles were able to deliver astaxanthin inside the cell, where it regulated gene expression. These nanoparticles showed antioxidant activity, and also minimal cytotoxicity on LX-2 cells, and could be useful in the prevention of liver fibrosis. However, part of the effect was due to the composition of the nanoparticles, since chitosan has a hepatoprotective effect *per se*. Bharathiraja et al. [137] reported the ability of astaxanthin-reduced gold nanoparticles to pass through the membrane and induce apoptosis in breast cancer cells MDA-MB-231 by the acceleration of the karyorrhexis (condensation of chromatin until it breaks). Nonetheless, these nanoparticles showed dose-dependent cytotoxicity that would limit their incorporation in food matrices.

The biological effect of astaxanthin-loaded particles has also been reported in vivo. Guan et al. [135] prepared astaxanthin-loaded nanoparticles of DNA and chitosan as those prepared by Wang et al. [128] and tested the antioxidant activity in Kunming male mice. The nanoparticles (288 nm size and positive charge) were intra-gastrically administered at different concentrations (0.3–0.9 mg of equivalent astaxanthin/kg), once a day for thirty days. Two animal groups administered with astaxanthin oil (0.9 mg/kg) or water were used as the control. Before sacrifice, an overdose of ethanol (50%) was administered to mice treated with astaxanthin to induce oxidative damage, observed as increments in the levels in plasma and liver of malondialdehyde (MDA) and protein carbonyl (PC) in plasma, and as a decrease in those of glutathione (GSH) and total SOD. The administration of nanoparticles did not show any adverse effects. The pretreatment of only 0.3 mg/kg of nanoparticles was enough to quench the oxidative damage and the results similar to those of the control group were observed in mice treated with 0.9 mg/kg. These results were not achieved when astaxanthin oil was administered, suggesting that the nanoencapsulation increased the bioavailability of astaxanthin. Simulated gastrointestinal digestion showed a decrease in the particle size in gastric and duodenum fluids, while it further increased in simulated jejunum fluid. The authors suggested that the presence of DNA in the nanoparticles facilitated the controlled release of astaxanthin in the duodenum. The nanoencapsulation greatly increased the bioavailability of astaxanthin, reaching maximum plasma levels after 4.12 h. This required around 1 h less than in the case of astaxanthin oil.

Chiu et al. [136] evaluated the hepatoprotective effect of astaxanthin-loaded nanoliposomes (240 nm) in Male Sprague-Dawley rats after a daily dose of free (10 mg/kg) or nano encapsulated astaxanthin (2–10 mg/kg) by gavage for 7 days. After the induction of acute hepatoxicity, they evaluated different parameters related to liver function and observed normal plasma levels of transaminases (GOT, GPT), creatinine, and blood urea nitrogen in mice gavaged with 10 mg/kg of nanoliposomes. The results improved those obtained with free astaxanthin, suggesting an increment in the bioavailability produced by the nanoencapsulation. Additionally, this dose of nanoliposomes induced anti-inflammatory activity by the regulation of the serum levels of nitrite, cytokine IL-6 and tumor necrosis factor-α (TNF-α). Moreover, it augmented the activity of antioxidant hepatic enzymes, and reduced the nuclear levels of the transcription factor NF-Κb (involved in inflammation and tumorigenesis), hence lowering the expression of induced nitric oxide synthase. This effect of 10 mg/kg of astaxanthin-loaded nanoliposomes was better than that of free astaxanthin. Moreover, 5 mg/kg of nanoliposomes were enough to decrease the infiltration of neutrophils in hepatic cells. These results suggest that the nanoliposomes were able to protect astaxanthin against gastrointestinal digestion, although the authors did not investigate if nanoliposomes released free astaxanthin in the gut or liver cells.

In another in vivo experiment, the oral administration of astaxanthin-loaded nanoliposomes (225 nm) for 4 weeks showed beneficial effects in wild-type C57BL/6J male mice with alcohol-induced liver fibrosis [125]. The oral administration of the nanoliposomes lowered the serum level of alanine aminotransferase (ALT). High serum levels of ALT levels indicated a damaged or inflamed liver, so nanoliposomes seemed to protect against alcohol-induced liver injury. Histopathological observations confirmed the reparative and protective effects of nanoliposomes in the liver, which suggests that astaxanthin reached the liver tissues. Although the authors did not compare the results obtained with those of oral administration of free astaxanthin, this work evidences an increment in the bioavailability of astaxanthin induced by nanoencapsulation in liposomes.

## 5. Limitations and Future Trends

Astaxanthin is economically the third most important carotenoid, after β-carotene and lutein. The market value of astaxanthin is expected to exceed 1.5 billion dollars by 2020 [138], with health and nutraceutical markets showing the fastest growth [26]. Encapsulation technologies are widely used in the pharmaceutical sector; however, their implementation in the food sector is more challenging. The body of literature accumulated on this topic suggests the high potential of encapsulation technologies to improve the functionality of astaxanthin and astaxanthin-containing lipid extracts for food applications, either as a techno-functional or a bioactive ingredient. However, it is to be noted that practical applications evidencing the antioxidant effect when applied to foods are scarce. The bioactive effect of the different astaxanthin loading-particles has been mainly demonstrated in primary cells or cell cultures, and the in vivo effect has just scarcely been demonstrated in rodents, and not at all in humans. In this regard, it is noteworthy that the demonstrated effect on one cell type cannot be generalized to any primary cell, and similarly, the results obtained with mice cannot be generalized to human beings.

Besides, many of these carriers have been developed recently and it is necessary to ascertain the concentration of particles necessary to exert a bioactive effect without showing toxicity. The interaction of the carriers with other components in the food matrix may negatively affect the stability, the release, and the bioavailability of astaxanthin as well, and therefore must be tested. It is also important to note that food processing conditions can alter the integrity of the astaxanthin loading-particles and dramatically affect bioavailability and bioactivity. The nature of food where the particles will be incorporated must be considered, as the presence of some food components (i.e., soluble dietary fiber) can harm the bioavailability of astaxanthin. It is important, then, to study the effect on stability and bioavailability of specific astaxanthin loading carriers individually, before and after incorporation in liquid and solid foods and further processing, and to evaluate the bioactivity in vitro and also in vivo using animal models. Adequate doses of astaxanthin-loaded particles and frequency of consumption must be established.

## Figures and Tables

**Table 1:** Improvement in technological properties or stability of astaxanthin and astaxanthin-containing lipid extracts (summary).

Astaxanthin Source	Encapsulation Method	Wall Material	Main Achievements	Reference
**Pure (≥98%), from Sigma Chemical Co.**	Inclusion complexation	β-cyclodextrin	Water solubility, heat, UV light, pH and oxidation stability	[66]
**Shrimp (*L. vannamei*) cephalothorax lipid extract**	Complex coacervation	Gelatin and gum Arabic or cashew gum	Astaxanthin stability (accelerated stability study)	[59,86]
**Purified esterified fraction from *H. pluvialis***	Complex coacervation	Whey protein and gum Arabic	Astaxanthin storage stability at different temperatures, illumination conditions, and atmospheres Increased release In vivo bioavailability	[53]
**Astaxanthin oleoresin (85% purity) from Shenyang Pharmaceutical Company**	Complex coacervation	Gelatin and alginate. Emulsifiers: Tween 80, soya lecithin or Span 20	Astaxanthin stability towards light, heat, and oxygen during storage In vivo bioavailability	[67]
**Shrimp (*L. vannamei*) cephalothorax lipid extract**	Spray-drying	Maltodextrin and/or gum Arabic Partially purified soya phosphatidylcholine (emulsifier)	Water solubility Astaxanthin, PUFAs and color stability (thermal treatment, chilled storage) Antioxidant activity Anti-inflammatory activity Bioaccessibility	[57,58]
**Oleoresin from *H. pluvialis***	Spray-drying	Gum Arabic or whey protein plus maltodextrin or inulin Soya lecithin (emulsifier)	Water solubility Temperature and pH stability dependent on wall composition (whey protein the highest stability)	[60]
**Oleoresin from *H. pluvialis***	Emulsification–solvent evaporation	Whey protein concentrate	Water solubility Stability towards UV light, thermal treatment, and Fe^3+^	[54]
**Extract from *P. rhodozyma***	Antisolvent precipitation	Zein and oligochitosan	Improved stability towards UV-light and storage	[48]
**Synthetic, from Sigma Chemical Co.**	Multiple emulsification–solvent evaporation	Chitosan (180 kDa molecular weight, 85.3% deacetylation degree) cross-linked with glutaraldehyde	No isomeration nor chemical degradation after 8 weeks of storage at 25–45 °C	[55]
**Pure (≥98%), from Sigma Chemical Co.**	Supercritical emulsions extraction	Ethylcellulose Tween 80 (emulsifier)	Antioxidant activity Release in intestinal fluid of 70% in 10 h	[102]
**Pure (≥97%), from Sigma Chemical Co.**	Multilayer micro-emulsification	Pectin from citrus peel Chitosan (1–20 kDa MW, 75% DD) Flaxseed oil (lipid carrier) Saponins (stabilizer)	Emulsion stability towards ionic strength and temperature Astaxanthin stability during storage	[82]
**Pure, from Sigma Chemical Co.**	Ionotropic gelation	Chitosan (low molecular weight, 82.6% deacetylation degree) Sodium caseinate Stearic acid	Aqueous dispersibility Antioxidant activity Anti-fibrinogenic activity (LX-2 cells)	[98]
**Nutraceutical grade astaxanthin, from Sigma Chemical Co.**	Modified emulsion gelation technology	Calcium alginate Paraffin oil (lipid carrier) Span 80 (Surfactant)	Water solubility Storage stability Antioxidant activity Cytostatic activity (adipose-derived stem cells)	[99]
**Astaxanthin-rich *X. dendrorhous* (AstaXin^®^, nutraceutical grade) from IGENE Biotechnology Inc.**	Ionotropic gelation	Calcium alginate	Antioxidant activity preserved during storage Release kinetics in gastrointestinal fluids regulated by modifying processing conditions	[70]
**Unknown, provided by Fuji Chemical Industry Co., Ltd. (Toyama, Japan)**	Polymer-coated solid lipid nanoparticles (hot homogenization method with sonication)	Bovine serum albumin-oxidized dextran complex (polymer coating) Precirol ATO 5 (lipid wall material and astaxanthin carrier)	Water solubility Antioxidant activity Retarded release in simulated gastrointestinal fluids	[68]
**Krill oil from *E. superba***	Nanostructured lipid carriers (hot homogenization method with sonication)	Palm stearin Lecithin (emulsifier)	Water dispersibility Nanoparticles stable to pasteurization and freeze-drying Enhanced stability of astaxanthin, DHA and EPA towards UV light	[56]
**Pure, from Sigma Chemical Co.**	Nanoliposomes	Egg phosphatidylcholine or dimyristoyl phosphatidylcholine	Water dispersibility Antioxidant activity in vitro Hydroxyl-radical cytotoxicity reduction in NIH-3T3 cells	[64]
**Shrimp lipid extract from *L. vannamei***	Nanoliposomes	Soya lecithin	Improved oxidative stability Better retention of EPA and DHA Reduction in fishy odor	[103]
**Pure (>96%), from Shanghai Yuanye Biotechnology Co.**	Nanoliposomes	Egg yolk lecithin Cholesterol (stabilizer) Lactoferrin and chitosan hydrochloride (external coatings)	Antioxidant activity improvement Stability improvement towards temperature or storage (external coatings provided additional protection) Improved bioaccessibility	[51]
**Extract from *P. rhodozyma***	Inclusion complexation	Carboxymethyl cellulose and microcrystalline cellulose	Improved solubility Improved antioxidant activity Improved stability towards temperature and acidic pH	[50]

**Table 2:** Functionality of encapsulated astaxanthin and astaxanthin-containing lipid extracts when applied to food products.

Astaxanthin Source	Encapsulation Method	Wall Material(s)	Food Product	Main Achievements	Reference
**Shrimp (*L. vannamei*) cephalothorax lipid extract**	Complex coacervation	Gelatin and gum Arabic or cashew gum	Gelled fish product Yogurt	Good dispersion and coloring functionality Masking characteristic shrimp odor in yogurt	[59,86]
**Shrimp (*L. vannamei*) cephalothorax lipid extract**	Spray-drying	Maltodextrin Partially purified soya phosphatidylcholine (emulsifier)	Gelatin gel Edible film	Good dispersion in gelatin gel and edible films Bioaccessibility increase	[57]
**Extract from *P. rhodozyma***	Antisolvent precipitation	Zein and oligochitosan	Apple and rice vinegar Liquor	Good dispersion and improvement of antioxidant activity	[48]
**Shrimp (*L. vannamei*) cephalothorax lipid extract**	Nanoliposomes entrapment	Soya phosphatidylcholine Glycerol (cryoprotectant)	Squid surimi-based product	Freeze-dried liposomes caused a slight decrease in gel strength but improved textural stability during frozen storage Homogeneous coloring Protection of astaxanthin from interaction with the gel matrix Prompting lipid oxidation during frozen storage	[87]
**Shrimp (*L. vannamei*) shells lipid extract**	Ultrasonic atomization	Alginate and chitosan	Yogurt	Good overall liking score (above 6 on the 9-point scale) Positive acceptance (86%) and purchase intent (95.6%)	[101]
**Extract from *P. rhodozyma***	Inclusion complexation	Carboxymethyl cellulose and microcrystalline cellulose	Yogurt	Good dispersion Improved antioxidant activity Improved stability of yogurt	[50]
**Shrimp (*L. vannamei*) hepatopancreas lipid extract**	Spray-drying	Sodium caseinate, gelatin, and glucose syrup	Biscuits	Good dispersion Good technological quality and sensory acceptance at an intermediate inclusion level Lower sensory impact than free astaxanthin Prompting lipid oxidation during storage	[97]
**Shrimp (*L. vannamei*) hepatopancreas lipid extract**	In situ β-glucan coated nanoliposomes	Lecithin β-glucan	Milk	Mild bitterness for nanoliposome-added milk Masking bitterness by coating with β-glucan (formed in situ when added to milk) No major quality changes after 15 days of refrigerated storage Viscosity increase in milk Presence of EPA and DHA in the bioaccessible fraction	[88]

**Table 3:** Bioactive functionality of encapsulated astaxanthin during in vitro and in vivo assays.

Particles Used to Load Astaxanthin	Bioactivity	Type of Essay	Reference
Nanoliposomes	Hepatoprotective	Mice	[125]
Chitosan-casein-oxidized-dextran nanoparticles	Antioxidant Prevention of liver fibrosis	LX-2 cells	[98]
DNA/chitosan nanoparticles	Cytoprotective Radical scavenging	Caco 2 cells Caco 2 cells	[128]
Nanoliposomes	Antiproliferative	HepG2 and Hep3B cells	[63]
Hyaluronan nanohydrogels	Antioxidant	HUVECs cells	[132]
Cyclodextrins	Antioxidant	HUVECs cells	[133]
Gold nanoparticles	Antiproliferative	MDA-MB-231 cells	[134]
DNA/chitosan nanoparticles	Antioxidant Hepatoprotective	Mice Mice	[135]
Nanoliposomes	Hepatoprotective	Mice	[136]
